# Insights into the protective immune response by immunization with full-length recombinant TprK protein: cellular and humoral responses

**DOI:** 10.1038/s41541-023-00748-1

**Published:** 2023-09-29

**Authors:** Dan Liu, Rui Chen, Yong-Jing Wang, Wei Li, Li-Li Liu, Li-Rong Lin, Tian-Ci Yang, Man-Li Tong

**Affiliations:** 1 Center of Clinical Laboratory, Zhongshan Hospital, School of Medicine, Xiamen University, Xiamen, China; 2 Institute of Infectious Disease, School of Medicine, Xiamen University, Xiamen, China

**Keywords:** Protein vaccines, Bacterial infection

## Abstract

Syphilis has resurged in many countries, which has called attention to vaccine development. Based on the immunization-based rabbit model of infection with the Nichols strain, this study explored the protective immune response of a controversial syphilis vaccine candidate, TprK, and found that immunization with full-length rTprK was effective in attenuating lesion development and accelerating lesion resolution, which could reduce the probability of the pathogen spreading to distant tissue sites to prevent the progression of the disease to some extent. Furthermore, the results revealed that immunization with rTprK not only rapidly induced a strong Th1-like cellular response but also elicited a humoral immune response to produce opsonic antibodies to enhance macrophage-mediated opsonophagocytosis. Although complete protection against infection was not achieved, the study provided a comprehensive and in-depth exploration of the immunogenicity of TprK and highlighted the importance of TprK as a promising syphilis vaccine component.

## Introduction

Syphilis is a systemic disease caused by *Treponema pallidum subsp. pallidum* (*T. pallidum*), threatening the health of people all over the world^1,2^. Although penicillin is effective for treatment, the incidence of syphilis has dramatically increased in many countries^3–5^. This has called attention to the urgent need for a vaccine for global containment strategies.

Indeed, since the Miller study using γ-irradiation to treat freshly isolated *T. pallidum* without affecting highly labile immunogenic antigens to achieve complete protection against the homologous strain of *T. pallidum*, the importance of antigens in *T. pallidum* as syphilis vaccine candidates has been highlighted^6^. Among them, the surfaced-exposed proteins have attracted attention, however, research has been greatly hampered by the incredibly slow growth of the pathogen and the paucity of surface-exposed proteins^7,8^. With the development of structural modeling of proteins, more information about putative surface-exposed proteins as candidate vaccinogens is being obtained^8,9^. Among them, TprK may be a controversial protein not only for the location of the proteins but also for its role in syphilis vaccine^10–12^. An early study using syphilitic infection experiments showed that immunization with a fragment (aa 37–348) of TprK attenuated syphilitic lesion development, and the variable domain was a target of opsonic antibody to promote phagocytosis^13^. However, the result was not reproduced in another laboratory, which used a 100-fold lower challenge dose but failed to observe any alteration of lesion development by immunization with variable domains of TprK, and the anti-TprK antibody did not significantly enhance phagocytosis^14^. Although subsequent research used the N-terminal portion (aa 37–273) of TprK for immunization, showing that immunization with the N-terminal portion of TprK retarded lesion development^15^, the protective immune response induced by immunization with TprK requires careful and comprehensive exploration.

Here, the full-length *tprK* gene of the standard Nichols strain, which was revealed to have almost a single *tprK* sequence^16,17^, was used as a template for the expression of recombinant TprK protein (rTprK). By employing immunized-infected rabbit models, we sought to fully investigate the role of TprK in the protective immune response against *T. pallidum* and revealed the induced cellular and humoral immune response, thus providing important information for understanding the immunogenicity of TprK.

## Results

### Immunization with rTprK significantly attenuated lesion progression and promoted lesion resolution

**Fig. 1:**
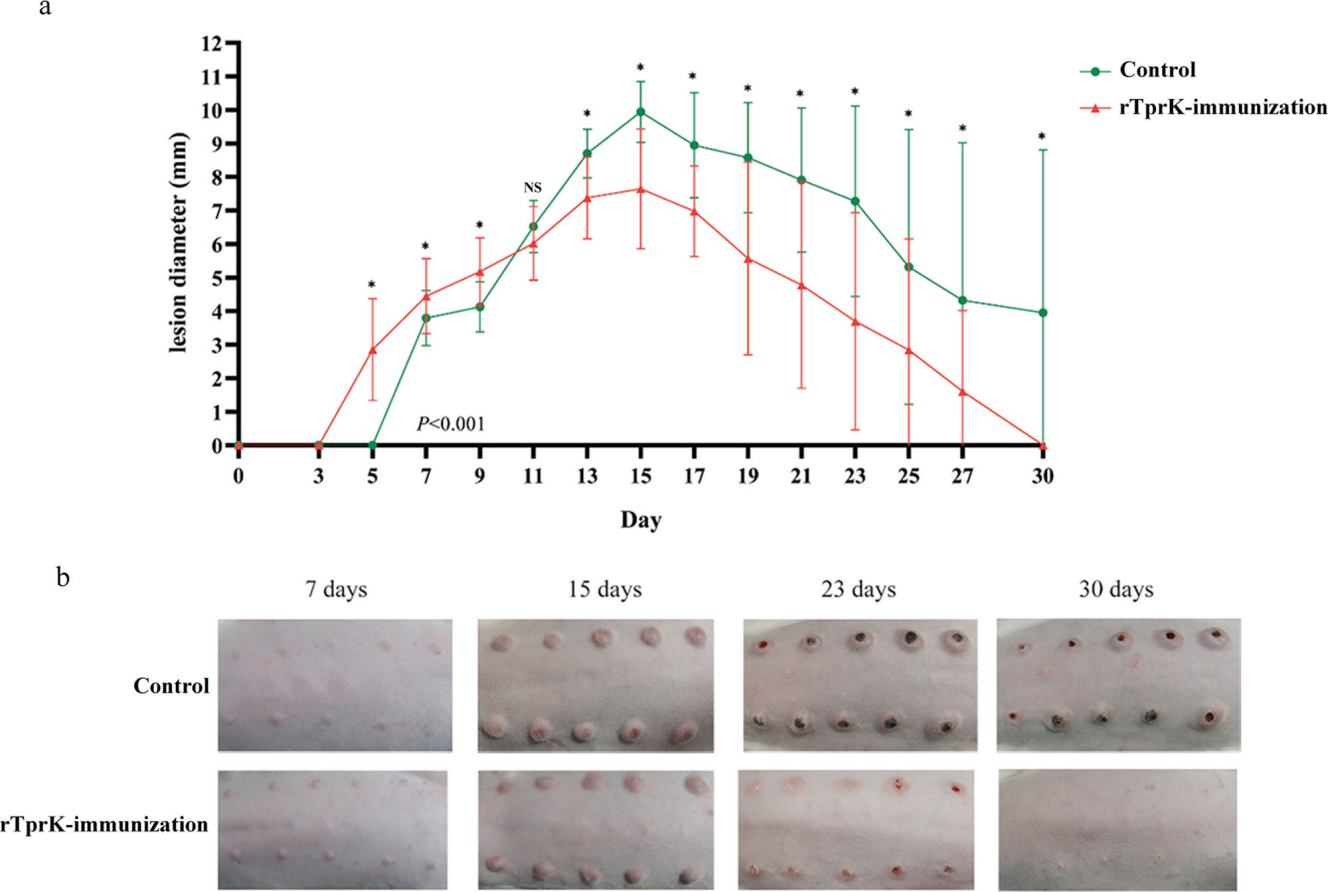
The development of lesions in the rTprK-immunized and control rabbits. **a** Dynamic lesion diameter changes in the rTprK-immunized and control rabbits. **b** Lesion development of representative rabbits at 7, 15, 23, and 30 days postchallenge in the two groups. The results are expressed as the mean ± SD. Repeated measures ANOVA was used to compare the overall diameter development of the lesion in the immunized and control rabbits, and Student’s *t* test was used to compare the mean diameter of lesions in the immunized and control rabbits (*P < 0.05; NS not significant).

After challenge with *T. pallidum*, the challenge sites of each rabbit were monitored. The immunized rabbits first developed small and measurable erythema at 3–5 days postchallenge, and the erythema became more obvious in the next two days, showing clinical evidence of a typical DTH response. For the control rabbits, a delayed and atypical DTH response appeared at 5–7 days postchallenge. Then, with the DTH response of the two groups resolved, the control lesions enlarged rapidly and developed typical chancres, and the mean diameter of lesions between the immunized and control rabbits gradually diverged at 7–15 days postchallenge. At 15 days postchallenge, the mean diameter of lesions in the immunized rabbits was significantly smaller than that in the control rabbits (7.65 ± 1.79 mm vs. 9.94 ± 0.91 mm, *P* < 0.05). Subsequently, the lesions of the two groups of rabbits gradually shrank. At 30 days postchallenge, all the lesions in the immunized rabbits were unmeasurable, while the control rabbits still had visible lesions, and the mean diameter of the lesions was 3.95 ± 4.86 mm. Significant differences in the overall diameter development of the lesion between the immunized and control rabbits were observed (*P* < 0.001, Fig. 1a). Representative photographs of lesion development are shown in Fig. 1b.

Indeed, some of the lesions in the immunized rabbits exhibited ulceration and induration after 15 days challenge with *T. pallidum*. The proportion of total lesions that progressed to ulceration was 42.0% (21/50) at 23 days postchallenge in the immunized rabbits. However, the lesions in the control rabbits were more severe, and 100% (50/50) of lesion sites in the control rabbits progressed to ulceration at 23 days postchallenge. There were significant differences in the proportion of lesions progressing to ulceration between the immunized and control rabbits (*P* < 0.001). At 30 days postchallenge, all the lesion sites in the immunized rabbits were completely resolved. The control rabbits still had ulceration proportion of 38.0% (19/50) and induration and resolution proportions of 8.0% (4/50) and 54.0% (27/50), respectively (Table 1). Overall, lesion development in the rTprK-immunized rabbits was significantly attenuated, and the resolution of the lesions was accelerated.

**Table 1:** Lesion data for rTprK-immunized and control groups.

	Postchallenge 23 days			Postchallenge 30 days		
	No. of ulcerative lesions (%)	No. of induration lesions (%)	No. of healed lesions (%)	No. of ulcerative lesions (%)	No. of induration lesions (%)	No. of healed lesions (%)
rTprK-immunized group	21/50 (42.0)	6/50 (12.0)	23/50 (46.0)	0/50 (0)	0/50 (0)	50/50 (100)
Control group	50/50 (100)	0/50 (0)	0/50 (0)	19/50 (38.0)	4/50 (8.0)	27/50 (54.0)
P* value	<0.001	<0.05	<0.001	<0.001	>0.05	<0.001

^*^The Chi-square test was used to compare rates of ulcerative, induration, and healed lesions between the rTprK-immunized and control group.

### Immunization with rTprK effectively reduced the *T. pallidum* burden in the challenge sites

**Fig. 2:**
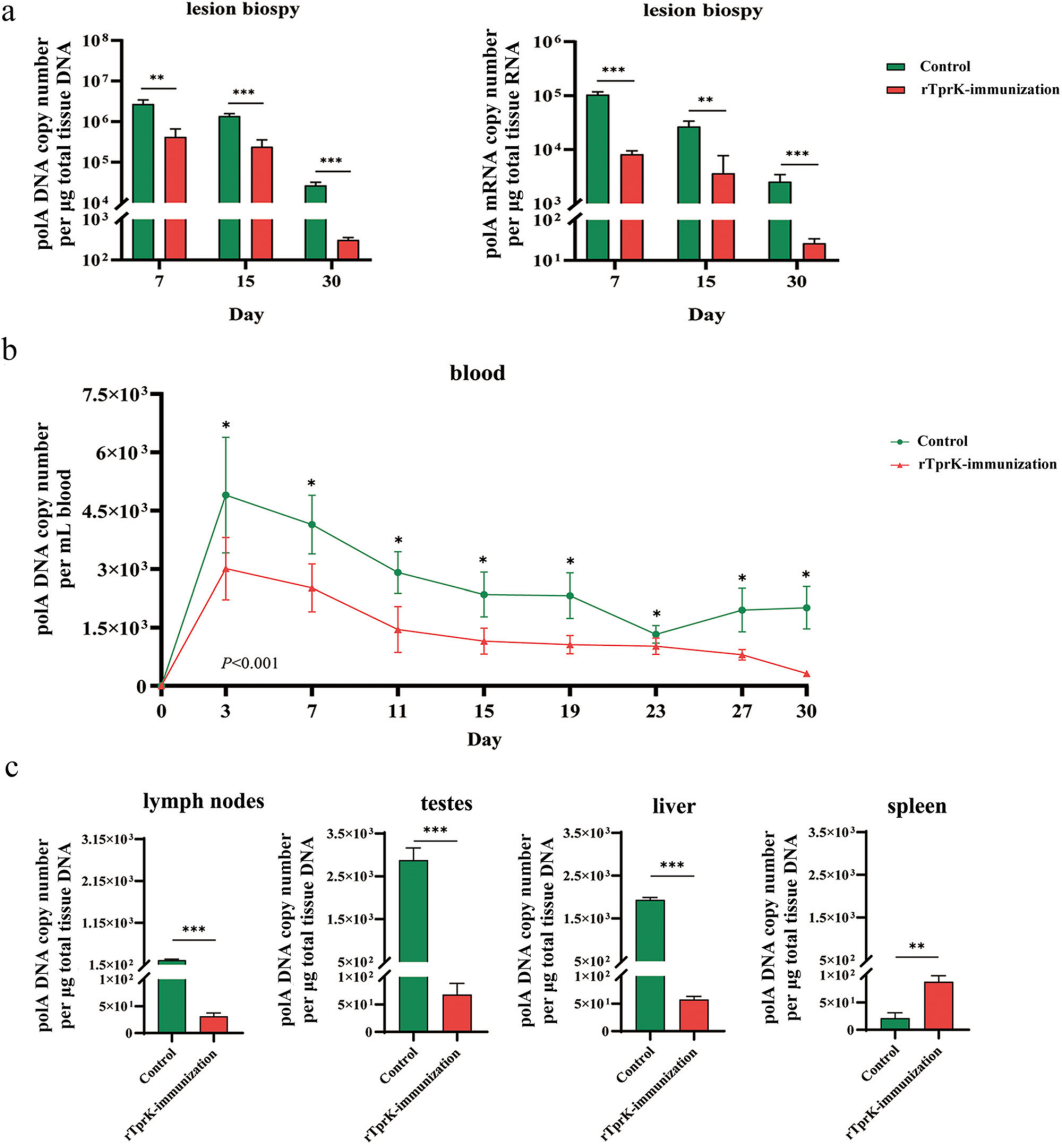
*T. pallidum* DNA in the tissues from the rTprK-immunized and control rabbits. **a** The *T. pallidum* burden of lesion biopsies was evaluated by targeting *polA*. **b** The disseminated burden in the blood was evaluated by targeting *polA*. **c** The disseminated burden in the tissues (lymph nodes, testis, liver, and spleen) was evaluated by targeting *polA*. The results were normalized to the total tissue DNA/RNA concentration, expressed as copies per μg of extracted tissue DNA/RNA. The *polA* in blood was expressed as copy number per mL of blood. The data are expressed as the mean ± SD. Student’s *t* test was used to compare the *T. pallidum* burdens in the different tissues of the two groups (*P < 0.05, **P < 0.01 ***P < 0.001).

To better assess the protective ability of immunization with rTprK, qPCR was performed to determine the *T. pallidum* burden in the two groups of rabbits. First, biopsy samples from lesions on days 7, 15, and 30 were analyzed. As shown in Fig. 2a, the average local *T. pallidum* DNA level in the rTprK-immunized rabbits was significantly decreased compared to that in the control rabbits at three time points (*P* < 0.001–0.01), and analysis of *T. pallidum* RNA yielded similar results (*P* < 0.001–0.01). Then, the *T. pallidum* load in the blood of all rabbits was measured every four days, and *T. pallidum* DNA could be detected in the blood of all rabbits over 30 days. Nevertheless, the overall trend for the *T. pallidum* burden in the bloodstream was reduced to a greater extent in the rTprK-immunized rabbits (*P* < 0.001), and the immunized rabbits exhibited less *T. pallidum* in the bloodstream at every time point (*P* < 0.05, Fig. 2b). Finally, the *T. pallidum* burden of distant organ sites of popliteal lymph nodes, testes, liver, and spleen was evaluated at 30 days postchallenge. The results showed that the lymph nodes, testes, and liver extracts in the immunized rabbits had lower *T. pallidum* burdens than those in the control rabbits (*P* < 0.001) (Fig. 2c). However, higher *T. pallidum* DNA was observed in the spleen of the immunized rabbits (*P* < 0.01), although the *T. pallidum* concentration in the two groups was slightly low.

**Fig. 3:**
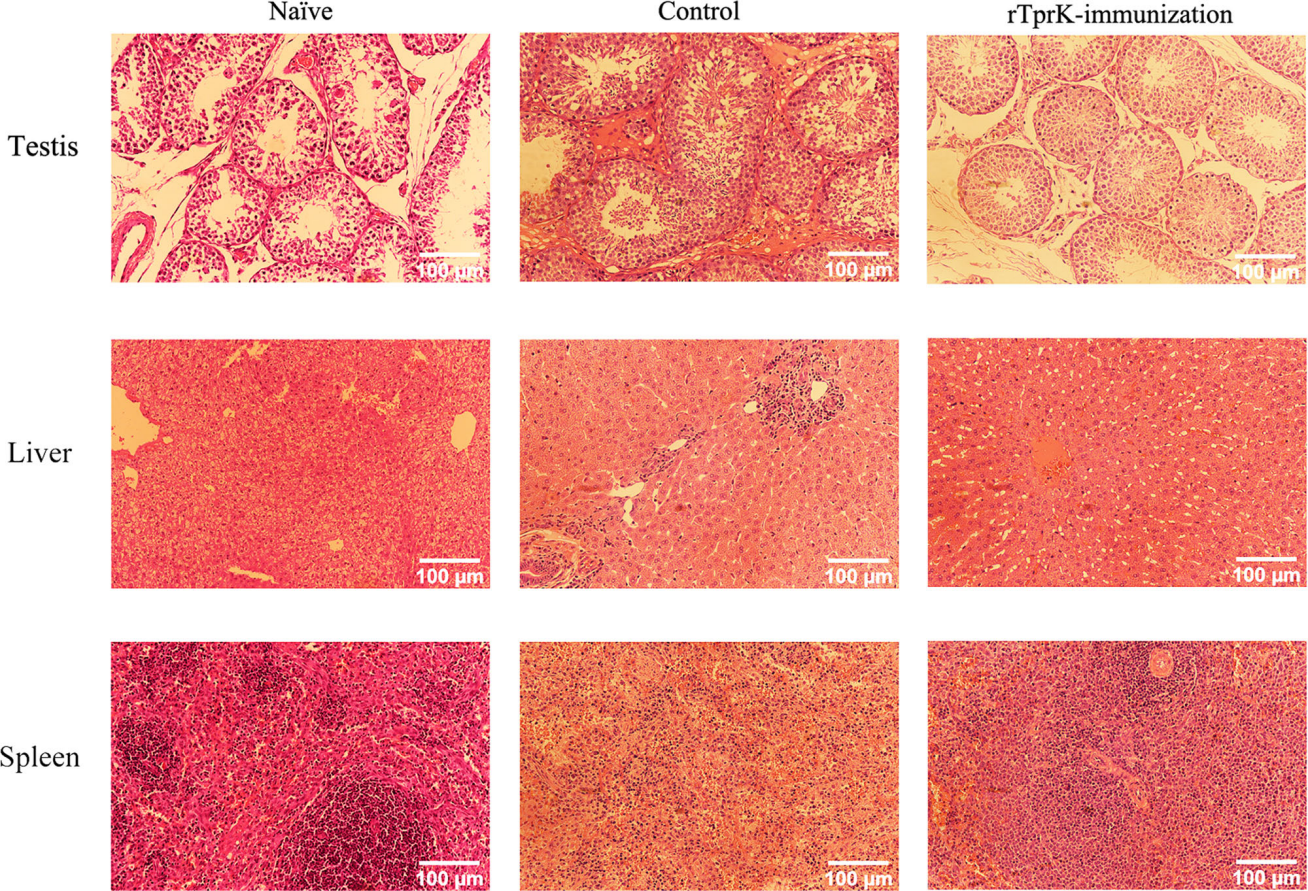
Histopathological changes in the testis, liver, and spleen at 30 days postchallenge. The naïve represented a control animal that was neither immunized nor challenged. Scale bars were shown as 100 μm.

In addition, hematoxylin and eosin (H&E) staining was used to visualize histopathological changes in the tissues at 30 days postchallenge. Examination of the testis, as an easily invaded organ, showed that seminiferous tubules in the control rabbits exhibited atrophy, irregular arrangement, and thickening of the interstation. The degree of inflammatory infiltration in the testis and liver of the rTprK-immunized rabbits was decreased. Notably, abundant immune cell infiltration was observed in the spleen of the immunized rabbits, while the cells in the control rabbits were relatively sparse (Fig. 3).

### Immunization with rTprK did not achieve complete protection against infection

Extracts of the popliteal lymph nodes of the rabbits in the two groups euthanized at 30 days postchallenge were transferred into serologically nonreactive recipient rabbits. All recipient animals showed seroconversion, indicating positive rabbit infectivity results, thus demonstrating that complete protection against infection by immunization with rTprK was not achieved; however, seroconversion of the recipient rabbits of the immunized groups occurred much later than that of the control groups (43.2 ± 3.3 days vs. 25.6 ± 2.2 days, *P* < 0.001). None of the recipient rabbits of the immunized groups develop orchitis, although there was no significant difference observed (0% (0/5) vs. 60% (3/5), *P* > 0.05). Besides, the recipient rabbits of the immunized groups showed less probability to be detected motile *T. pallidum* in the dark-field microscopy than the recipient rabbits of the control groups (20% (1/5) vs.100% (5/5), *P* < 0.05) (Supplementary Table 1).

### rTprK immunization enhanced Th1-like cytokine production

**Fig. 4:**
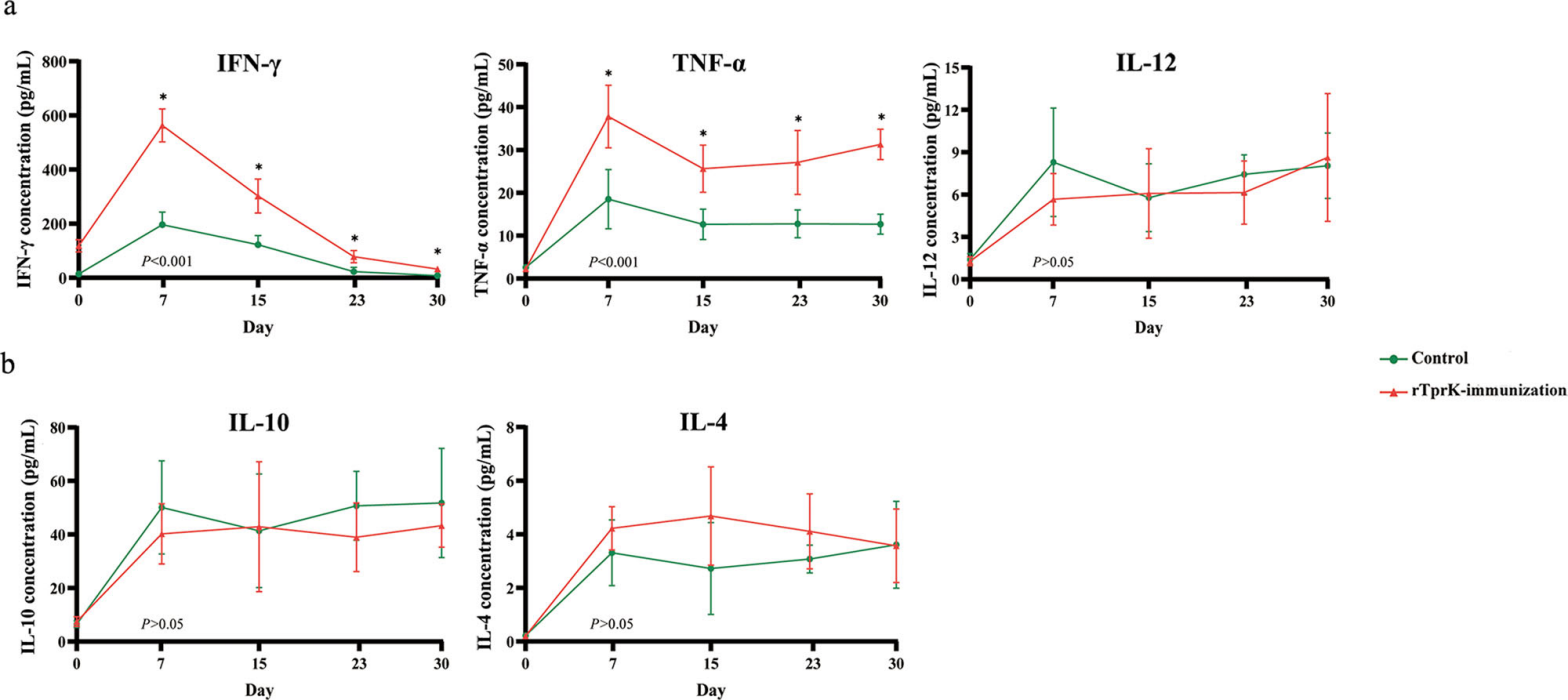
The secretion of Th1-like cytokines and Th2-like cytokines in the serum of the rTprK-immunized and control rabbits. **a** The levels of Th1-like cytokines (IFN-γ, TNF-α, and IL-12) in the serum were evaluated by ELISA. **b** The levels of Th2-like cytokines (IL-10 and IL-4) in the serum were evaluated by ELISA. The results are expressed as the mean ± SD. The overall expression levels were compared using repeated-measures ANOVA, and the expression levels at specific time points were compared using Student’s *t* test (*P < 0.05).

To evaluate the cellular immune response induced by rTprK, the levels of cytokines in the serum of all challenged rabbits were monitored. The overall trends of the expression levels of the Th1-like cytokines IFN-γ, TNF-α, and IL-12 were significantly higher in the rTprK-immunized rabbits than in the control rabbits (*P* < 0.001). Their expression was significantly different at 7, 15, 23, and 30 days postchallenge (*P* < 0.05), especially at 7 days postchallenge, when their secretion was robustly increased (Fig. 4a). However, the expression levels of the Th2-like cytokines IL-10 and IL-4 were not significantly different between the two groups (Fig. 4b).

**Fig. 5:**
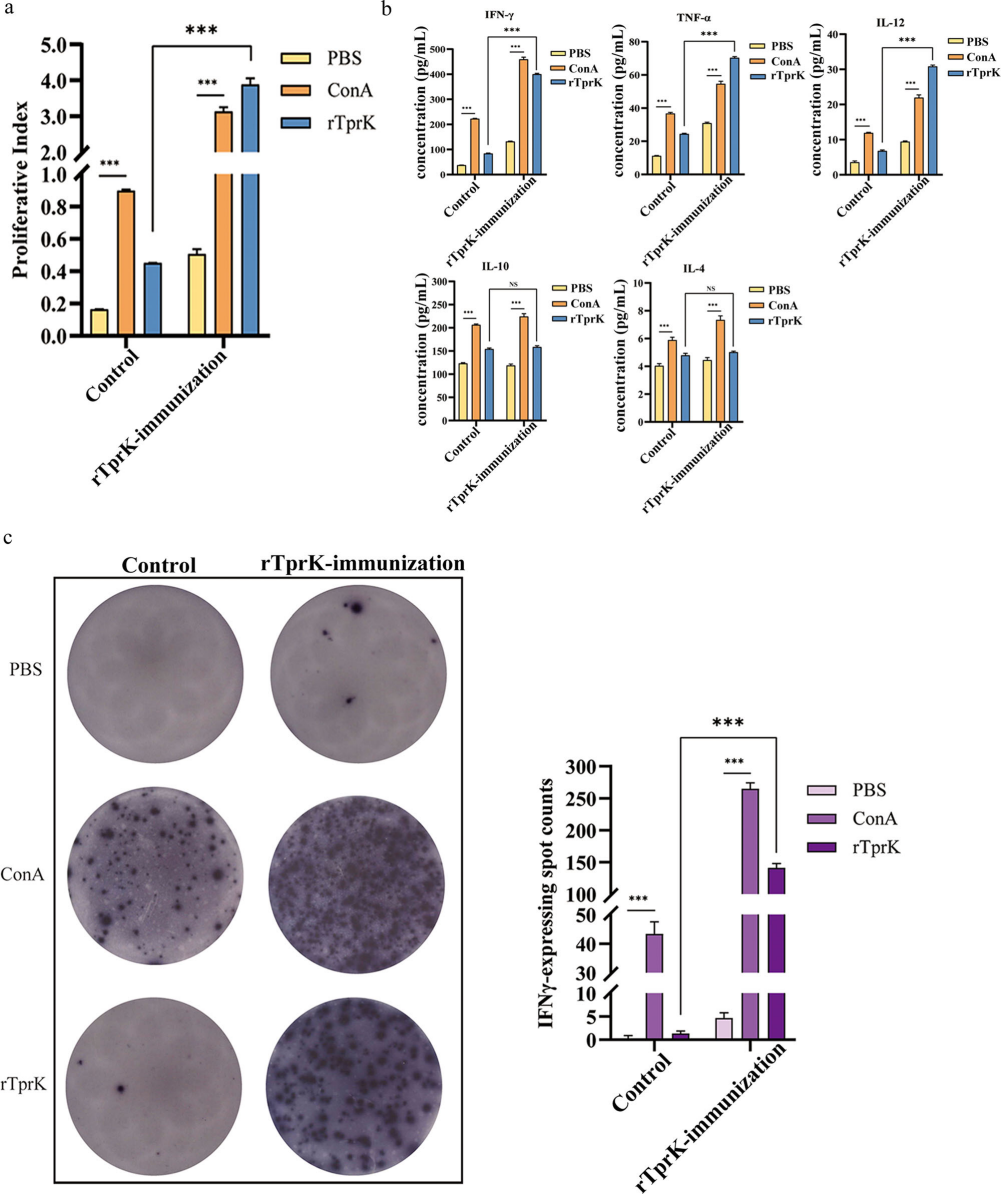
T-cell responses in vitro. **a** T-cell proliferation by rTprK stimulation. **b** The levels of IFN-γ, TNF-α, IL-12, IL-10, and IL-4 by rTprK stimulation of PBMCs. **c** The counts of IFN-γ spot-forming cells. The results are expressed as the mean ± SD, and Student’s *t* test was used to compare the levels of cytokines between the two groups (***P < 0.001; NS not significant).

Meanwhile, the role of rTprK in inducing the T-cell response was also confirmed in vitro. Before *T. pallidum* challenge, peripheral blood mononuclear cells (PBMCs) were isolated from the immunized and control rabbits, and rTprK was applied to stimulate PBMCs. Higher proliferation of T cells was found in the cells from the immunized rabbits (Fig. 5a). Consistent with the observation of increased expression of the Th1-like cytokines IFN-γ, TNF-α and IL-12 in the rTprK-immunized rabbits after challenge with *T. pallidum*, their expression was significantly higher after stimulation with rTprK (*P* < 0.001). For the secretions of the Th2-like cytokines IL-10 and IL-4, there were also no significant differences found between the two groups (Fig. 5b). In addition, specific T-cell responses quantified with IFN-γ were performed, and a significant T-cell response of IFNγ-ELISpot was observed in the rTprK-immunized group compared to the control group (*P* < 0.001, Fig. 5c).

### Sera from the rTprK-immunized rabbits significantly facilitated macrophage-mediated opsonophagocytosis

To investigate the effect of rTprK-induced humoral immunity on *T. pallidum*, opsonophagocytosis assays were performed in vitro. The prechallenge sera were obtained from the two groups of rabbits, in which the immunized rabbits had an anti-rTprK antibody titer of 1:200,000 and the control rabbits had non-detectable anti-rTprK antibody titer. In addition, the postchallenge sera (at 30 days postchallenge) were also obtained from the two groups of rabbits, in which the immunized rabbits maintained a steady high anti-rTprK antibody titer (higher than 1:204,800); in contrast, the control rabbits only produced a low level of anti-rTprK antibody titer (lower than 1:6400) (Supplementary Fig. 1).

**Fig. 6:**
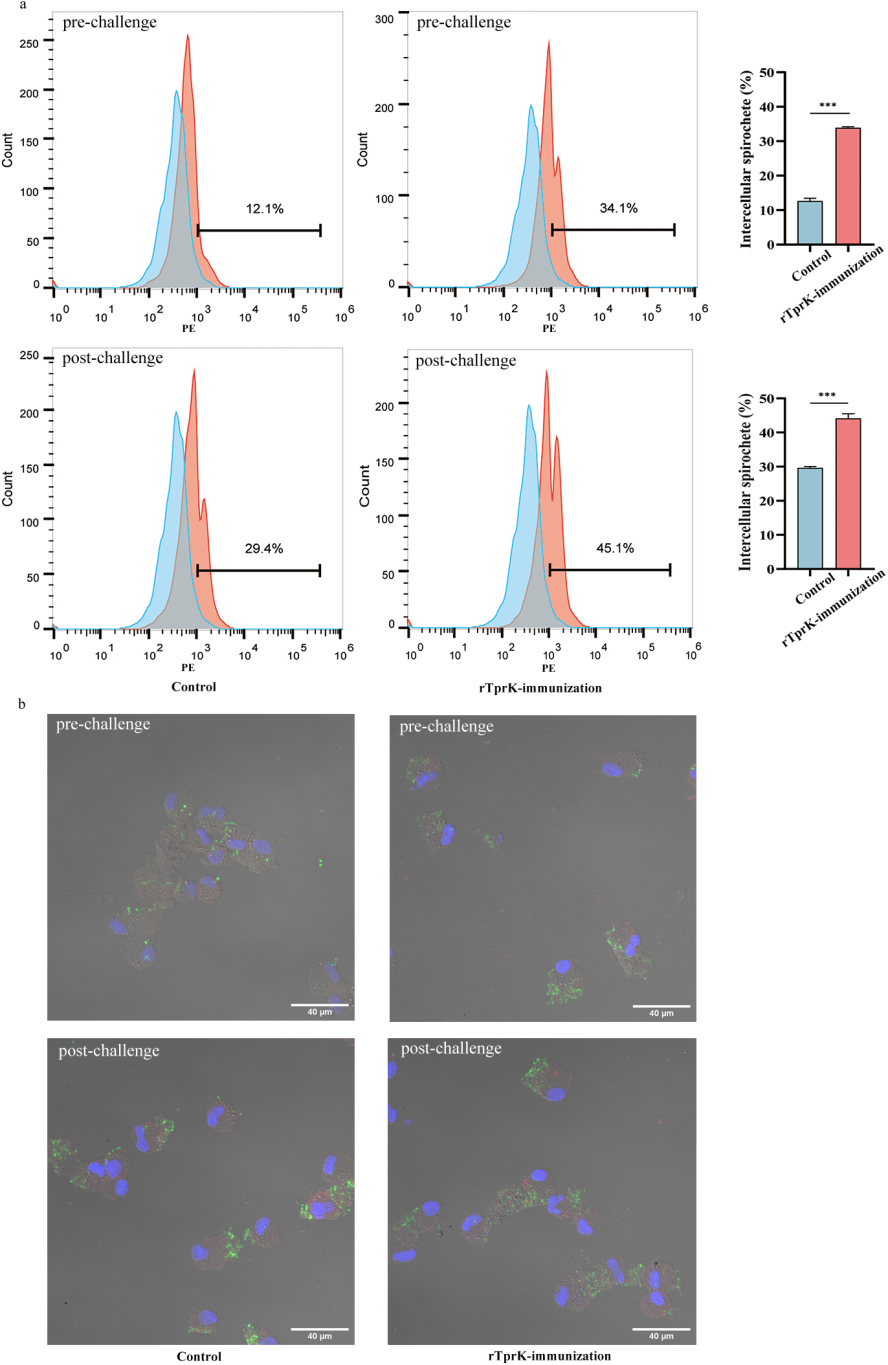
Phagocytosis of macrophages was determined by flow cytometry and indirect immunofluorescence analysis. **a** Flow cytometry histograms representing the intracellular spirochete burden. **b** Spirochete internalization by macrophages was stained green. The nuclei and actin filaments of macrophages were stained with DAPI (blue) and phalloidin (red). Then, the profiles of macrophages were captured under the bright field of the microscope. Scale bars were shown as 40 μm. The data are expressed as the mean ± SD. Student’s *t* test was used to compare the means of intracellular spirochetes between the two (***P < 0.001).

First, prechallenge rabbit sera from the above-described rTprK-immunized and control rabbits were used for culture to assess their opsonic ability. Flow cytometry analysis showed that the sera from the immunized rabbits significantly promoted *T. pallidum* ingestion compared to the control (*P* < 0.001, Fig. 6a and Supplementary Fig. 2). Additionally, sera from the immunized and control group rabbits at 30 days postchallenge were used in the opsonophagocytosis assay. As expected, a significant increase in macrophage phagocytosis of *T. pallidum* was observed in the presence of sera from the immunized rabbits (*P* < 0.001, Fig. 6a and Supplementary Fig. 2). Moreover, indirect immunofluorescence microscopy was performed to observe the phagocytosis of macrophages. The results showed that the phagocytosis of macrophages was greatly enhanced in the presence of the prechallenge sera from the immunized rabbits, and more *T. pallidum* was ingested when the sera from the immunized rabbits 30 days postchallenge were applied (Fig. 6b and Supplementary Fig. 3), which was consistent with the results of flow cytometry analysis.

## Discussion

TprK is not only considered to have high variability in promoting *T. pallidum* evasion but also has marked immunogenicity^8^. In this study, rTprK-immunized rabbit models of infection by the Nichols strain were constructed. Obvious attenuation of lesion development and lower *T. pallidum* load in the tissue sites were observed in the rTprK-immunized rabbits, which was consistent with previous findings^13,15,18^. Furthermore, immunization with rTprK elicited a robust Th1-cell response led by high expression of IFN-γ. Meanwhile, the humoral response induced by rTprK in the presence of opsonic antibodies enhanced macrophage-mediated opsonophagocytosis. Our results highlighted the importance of TprK as a syphilis vaccine component.

Generally, syphilis transmission occurs by contact with infectious lesions^19^, and immunization with rTprK was effective in attenuating lesion progression and accelerating lesion resolution, which might mean that the probability of syphilis transmission to sex partners would be reduced. Subsequent monitoring of the *T. pallidum* burden in the bloodstream and disseminated tissues showed a significantly lower total *T. pallidum* burden in the rTprK-immunized rabbits, illustrating that rTprK immunization facilitated the host clearance of *T. pallidum* at primary lesion sites and reduced the probability of the pathogen spreading to distant tissue sites. It should be noted that the number of *T. pallidum* burden in the blood was not very high, and persistent low-level spread to disseminated tissue sites was likely to occur. With prolongation of the infection time, *T. pallidum* might gradually accumulate in the tissues and cause tissue damage, which was supported by the observed tissue histopathological changes. Moreover, lymph node transfer in this study was conducted on day 30 postchallenge. Although the rabbit infectivity test (RIT) of the two groups was positive, the recipient rabbits of the immunized groups did not develop orchitis and took a longer time to seroconvert, which indicated that immunization with rTprK quickly mobilized the immune reaction and executed the clearance of pathogens at primary sites to prevent the progression of the disease to some extent.

Based on the examination of histopathology of primary lesions in human syphilis infection as well as rabbit infection models, the production of Th1-skewed cytokines to induce a strong DTH response was critical for limiting the number of *T. pallidum*^20–22^. In the study, within 7 days postchallenge, a typical DTH response was observed in the rTprK-immunized rabbits. Meanwhile, the levels of the Th1-like cytokines IFN-γ, TNF-α, and IL-12 were significantly increased in the sera of rTprK-immunized rabbits, and at 7 days postchallenge, the secretion of these cytokines peaked. IFN-γ is a marker of the DTH response, and the significantly increased expression of this marker corroborated the occurrence of a typical DTH response in the rTprK-immunized rabbits. There was also some indirect evidence of abundant immune cell infiltration in the spleen of the rTprK-immunized rabbits, suggesting that the cellular immune response in the immunized rabbits was intense. In combination with the significantly reduced number of *T. pallidum* in lesions of the immunized rabbits, the results indicated that immunized rTprK could rapidly evoke the host to robustly activate the Th1 immune response to limit the number of *T. pallidum* in lesions, thus altering lesion development^19,23^.

The humoral response is thought to be important in the clearance of *T. pallidum* due to the presence of opsonic antibodies that facilitate the ingestion and killing of *T. pallidum* by macrophages^24,25^. However, whether TprK is a target of the opsonic antibodies has been a subject of debate between two laboratories^13,14^. They both applied rabbit peritoneal macrophages incubated with *T. pallidum* in the presence of rabbit anti-TprK antisera, which was directed to the purified recombinant variable domain of rTprK, but their findings were opposite, with one demonstrating that the anti-TprK antisera could opsonize *T. pallidum* (Nichols strain) for phagocytosis and the other demonstrating that the anti-TprK antisera did not promote phagocytosis. The reason is still unclear. Given the current diversity and sophistication of research tools, we applied flow cytometry analysis and indirect immunofluorescence microscopy to analyze the role of anti-rTprK sera. The opsonophagocytosis results showed that macrophage-mediated opsonization of *T. pallidum* with prechallenge sera from the rTprK-immunized rabbits led to a higher phagocytosis rate. Furthermore, with postchallenge sera from the immunized rabbits, macrophage-mediated opsonization of *T. pallidum* was significantly strengthened. The findings proved that antibodies against rTprK could enhance opsonophagocytosis of *T. pallidum*.

Although this study exhibited the whole immune response by rTprK immunization, it should be noted that the exploration of Th1-cell responses is still insufficient. More research on the Th1 cell responses induced by TprK needs to be performed. More importantly, the study did not further investigate the epitopes directed to the T-cell response and B-cell response, which would shed light on the development of effective measures to cope with clinical *T. pallidum* strains with highly variable *tprK* genes. In addition, it would be better to prolong the observation period, more information could be obtained which might help to understand the battle between *T. pallidum* and host immune response. And the study could not explain why more *T. pallidum* was detected in the spleen of the immunized rabbits and whether it was related to abundant immune cell infiltration in the spleen of the immunized rabbits. More work is needed to determine the reason.

In summary, the immunogenicity of full-length rTprK against *T. pallidum* was effective in attenuating lesion progression and reducing the *T. pallidum* burden. Immunized rTprK not only induced a strong Th1-like cellular response but also produced opsonic antibodies to enhance macrophage-mediated opsonophagocytosis. Although complete protection against infection was not achieved in this study, the study provided a comprehensive and in-depth exploration of the immunogenicity of TprK and highlighted the importance of TprK as a promising syphilis vaccine component. Moreover, the study provides a good foundation for further investigation of the effect of variations in TprK on the immune response.

## Methods

### Ethics statement

This study was approved by the Institutional Ethics Committee of Zhongshan Hospital of Xiamen University, School of Medicine, Xiamen University. All rabbit experimental protocols strictly followed the parameters outlined by the Institutional Animal Care and Use Committee and were approved by the Animal Experimental Ethics Committee of the School of Medicine, Xiamen University.

### Preparation of *T. Pallidum*

The *T. pallidum* Nichols Seattle strain was kindly provided by Lorenzo Giacani, Ph.D. (University of Washington, Seattle), and the frozen spirochete was propagated intratesticularly in New Zealand White rabbits and the Nichols strain was harvested at peak orchitis^26^.The strain was propagated for three rapid passages and used for subsequent challenge experiments.

### Recombinant expression of full-length TprK protein

DNA extraction was directly from the residuals of the frozen spirochete suspension and full-length *tprK* gene amplification was performed using the following primers: sense 5′- ATGGGTCGCGGCGAATTCATGATTGACCCATCTG- 3′ and antisense 5′- GTGGTGGTGGTGGTGCTCGAGCTACCAAATCAAGCGAC- 3′^27^. The amplicon was cloned into the pET28a vector (Miaoling, Wuhan, China) with an N-terminal 6×His tag between the EcoRI and XhoI sites and transformed into the *E. coli* expression strain BL21(DE3) (Tiangen, Beijing, China) according to the manufacturer’s instructions.

Protein expression was performed in LB medium supplemented with 0.3 mM IPTG and 100 μg/mL of kanamycin at 16 °C for 20 h. Inclusion bodies containing the recombinant proteins were determined by SDS-PAGE. For recombinant TprK protein purification, *E. coli* cells were pelleted by centrifugation at 6000×g for 10 min, 4 °C and lysed in buffer A (50 mM Tris−HCl, pH 8.0, 1 mM EDTA, 50 mM NaCl, 1% Triton X-100, 1% 0.1 M PMSF, 0.1% β-ME). Then cell suspensions were collected by centrifugation at 12,000×g for 20 min 4 °C and resuspended in buffer B (8 M Urea, 0.1 M Tris−HCl, pH 8.0). Resulting samples were loaded onto affinity chromatography columns loaded with Ni-nitrilotriacetic acid (Ni-NTA) agarose (Qiagen, Shanghai, China). The eluted step was performed with 10 mL elution buffer (0.5 M NaCl, 20 mM Tris−HCl, pH 8.0, 1 M imidazole, 6 M Urea). By SDS-PAGE analysis and Coomassie blue staining, the elute fractions were determined whether contained contaminating proteins. Then the fractions were dialyzed for 48 h at 4 °C with the appropriate molecular weight cut-off against 1× phosphate-buffered saline (PBS) containing gradually decreasing concentrations of elution buffer and concentrated using a spin concentrator with further filtering 15 kDa proteins (Millipore, Massachusetts, USA). After dialysis, soluble proteins were analyzed by western blot using anti-His tag antibody, sera from rabbits infected with *T. pallidum* Nichols strain. Moreover, the sera from rTprK-immunized rabbits were used to react with Nichols whole cell lysates to further confirm the availability of the purified rTprK. All blots were processed in parallel and derive from the same experiments (Supplementary Fig. 4). After the endotoxin was removed with an EtEraser™ Endotoxin Removal Kit (Chinese Horseshoe Crab Reagent Manufactory, Ltd., Xiamen, China)^28^, rTprK was stored at -80°C until use.

### rTprK immunization and *T. pallidum* challenge procedure

Outbred Male New Zealand White rabbits (3.5 kg, 13–15 weeks of age, Xiamen University Laboratory Animal Center, Xiamen, China) with negative Venereal Disease Research Laboratory (VDRL) test and *T. pallidum* particle agglutination (TPPA) test were randomly selected in this study. Five rabbits were immunized with 500 μg of rTprK in an aqueous buffer with TiterMax Gold adjuvant (Sigma, St Louis, MO, USA) at a 1:1 ratio. Immunizations were administered subcutaneously (250 μg) and intramuscularly (250 μg) three times at two-week intervals. Five control rabbits administered PBS were used under the same administration conditions. All rabbits were fed antibiotic-free food and water, and were housed at 18–20°C.

At 10 days after the final immunization (anti-TprK antibody titer reached 1:200,000 in the rTprK-immunized rabbits), all rTprK-immunized rabbits and control rabbits were anaesthetized with acepromazine via intramuscular injection at 1 to 3 mg/kg body weight and challenged intradermally with 0.1 mL of 1 × 10^7^ /mL fresh *T. pallidum* (Nichols strain) at 10 sites on their shaved backs. Challenge sites were monitored for erythema, induration, and ulceration and measured every two days to assess lesion diameter. At 7, 15, and 30 days postchallenge, the two sets of skin lesions were biopsied (4-mm punch) from three rabbits in each group for *T. pallidum* DNA and RNA measurement and histological analysis. The remaining two rabbits in each group were only used for the observation. One milliliter of blood was collected every 4 days for measurement of *T. pallidum* DNA, cytokine secretion, and the serum antibody response and for the opsonophagocytosis assay. At day 30 postchallenge, the challenge sites of rTprK-immunized rabbits had resolved, all rabbits were euthanized with pentobarbital via intravenous injection at 90 mg/kg body weight, and the liver, spleen, testes, and popliteal lymph nodes were removed for *T. pallidum* DNA measurement and histological analysis. The popliteal lymph nodes were also transferred to the testes of randomly assigned naïve rabbits to observe infectivity^29^. A positive RIT was considered if seroconversion appeared, and/or orchitis developed, and/or motile *T. pallidum* was observed under dark-field microscopy in the recipient animals. After a three-month observation, the negative RIT animals were euthanized, and their popliteal lymph nodes were transferred to a new naïve rabbit again (Supplementary Fig. 5). The RIT result was finally determined after a new three-month observation period. In addition, another two rabbits only administered TiterMax Gold adjuvant were parallelly challenged by *T. pallidum*, and they showed similar results to the control rabbits; thus, the influence of adjuvant in the immunization was ruled out.

### *T. pallidum* quantitation by qPCR

DNA was extracted from the lesions/tissues/blood using a DNeasy Blood and Tissue Kit (Qiagen, Shanghai, China) according to the protocol recommended by the manufacturer, and careful precautions were implemented to avoid DNA cross-contamination^30^. Total RNA from the lesions was isolated using the RNeasy Kit (Tiangen, Beijing, China) and was then treated with DNase I (Invitrogen, Carlsbad, CA). The concentration of DNA/RNA was obtained from spectrophotometric measurements. Reverse transcription of RNA was performed using a high-capacity cDNA reverse transcription kit (Takara, Kyoto, Japan). The DNA/cDNA samples were quantified by targeting *polA* through quantitative real-time PCR (qPCR) using a 96-well reaction plate with a LightCycle 480 system (Roche, Basel, Switzerland). The primers used for targeting *polA* were as follow: sense 5′- TACGGTGCAAGTGCTCAGAC- 3′ and antisense 5′- CAGGCACATTGTCGGAGGAA- 3′. A standard curve was constructed using 10-fold serial dilutions of linearized plasmid (for *polA*) DNA to calculate the *polA* copies. The results were normalized to the total tissue DNA/RNA concentration, expressed as copies per μg of extracted tissue DNA/RNA. The *polA* in blood was expressed as copy number per mL of blood^31^.

### Histology

Samples taken from the lesions, livers, spleens, and testicles were fixed in formalin, mounted, bisected, vertically sectioned at 4 μm and processed for H&E staining to observe the cellular infiltration and structural changes in tissues. Meanwhile, a naïve control animal that was neither immunized nor challenged was also include to analyzed.

### Analysis of cytokine secretion in the sera after challenge with *T. pallidum*

Blood samples were obtained via ear bleeds from all rTprK-immunized and control rabbits every four days. The expression of Th1-like cytokines (IFN-γ, TNF-α, and IL-12) and Th2-like cytokines (IL-10 and IL-4) in the sera was measured using ELISA kits (Jianglai, Shanghai, China) according to the manufacturer’s instructions.

### Evaluation of T-cell responses

At 10 days after the last rTprK booster, that is, before challenge with *T. pallidum*, all rTprK-immunized and control rabbits were bled for analysis of the T-cell response in vitro. PBMCs were isolated from all obtained blood samples. A methyl thiazolyl tetrazolium (MTT) assay was used to detect T-cell proliferation. Briefly, approximately 10^5^ cells in 200 μL of culture medium were plated in triplicate wells of 96-well plates. rTprK (2.5 μg) was added and incubated at 37 °C in a 5% CO_2_ atmosphere. Concanavalin A (ConA, 0.5 μg/well, ICN Pharmaceuticals, Costa Mesa, CA, USA) was used as the positive control, and PBS was used to measure background reactivity. After 48 h of incubation, 50 μg MTT was added to each well and incubated for 4 h. Then, 150 μL of dimethyl sulfoxide (DMSO) was added, and the absorbance was measured at 490 nm. In parallel, 48 h after exposure to rTprK, ConA, or PBS, the supernatants of cells were also collected to detect the secretion of Th1-like cytokines (IFN-γ, TNF-α, and IL-12) and Th2-like cytokines (IL-10 and IL-4) using ELISA kits as mentioned above.

In addition, specific T-cell responses quantification with IFN-γ was performed using an ELISpot kit (Mabtech, Stockholm, Sweden) according to the manufacturer’s protocol. Briefly, approximately 10^5^ PMBCs were plated on ELISpot M96-well plates and incubated with 2.5 μg rTprK for 48 h. ConA (0.5 μg/well) and PBS were used as the positive and negative controls, respectively. Spot-forming cells were counted using a Cellular Technology Limited S6 Universal Analyzer (Cellular Technology, Cleveland, Ohio, USA), and data were processed with ImmunoSpot®7.0 software.

### Monitoring the titer of anti-rTprK in the sera after challenge with *T. pallidum*

The antibody titer against rTprK was monitored with an enzyme-linked immunosorbent assay^29^. In brief, 50 μL of 1 μg/mL rTprK was used as the coating antigen and incubated at 4 °C overnight. A goat anti-rabbit IgG H&L antibody (ab6721, Abcam, Cambridge, UK) was used as a secondary antibody at a 1:10,000 dilution, and the absorbance was measured at 450 nm. The cutoff value was 2.1 times the mean value of the negative sample. Individual sera were tested in triplicate wells per serum sample.

### Opsonophagocytosis assay

Proteose peptone-induced rabbit peritoneal macrophages were isolated from normal rabbits with negative VDRL test and TPPA test^32^. Serum samples from the rTprK-immunized and control rabbits at prechallenge (10 days after the last rTprK booster) and postchallenge (30 days postchallenge) were used to assess opsonization capacity. In brief, macrophages were cultured in DMEM medium with 10% serum (heat inactivated at 56 °C for 30 min), and fresh *T. pallidum* was added at an MOI of 10:1 for 12 h of incubation. Fluorescence-activated cell sorting (FACS) analysis was used to evaluate *T. pallidum* internalization by macrophages^33^. Anti-Tp47 mouse monoclonal antibody at a 1:50 dilution was used, which was generated by the Boson Biotech company (Xiamen, China). And phycoerythrin (PE)-conjugated anti-mouse secondary antibody at a 1:100 dilution (ab150116, Abcam, Cambridge, UK) was used. Counts of PE-labeled cells determined by FACS Canto II flow cytometer (BD, New Jersey, USA) represented the percentage of internalized spirochetes. Meanwhile, immunofluorescence microscopy was performed using the anti-Tp47 mouse monoclonal antibody and FITC-conjugated goat anti-mouse IgG (ab150113, Abcam, Cambridge, UK). Fluorescence images were captured by a laser scanning confocal microscope (Zeiss Axio LSM 880, Germany)^33^.

### Statistical analysis

All statistical analyses were performed using SPSS version 19.0 (SPSS, Chicago, IL, USA). Quantitative materials were reported as the mean ± SD. Differences between the two groups were analyzed by Student’s *t* test and repeated-measures ANOVA. Qualitative materials were used in the Chi-square test to identify significant differences between the groups. A two-sided *P* < 0.05 was considered statistically significant.

### Supplementary information

Supplementary Information

reporting-summary

## Data Availability

The sequence of full-length *tprK* gene used for recombinant protein product was deposited in GenBank under accession number OR233723. The authors declare that all data supporting the findings of this study are available within the paper and its supplementary information files.
