# Supplementary material for: Insights into the protective immune response by immunization with full-length recombinant TprK protein: cellular and humoral responses
Source: NPJ Vaccines. 2023 Sep 29;8:146. doi: 10.1038/s41541-023-00748-1 (PMC10542339; doi:10.1038/s41541-023-00748-1)
Supplement: Supplementary file 1 — Supplementary Information [file 41541_2023_748_MOESM1_ESM.pdf]

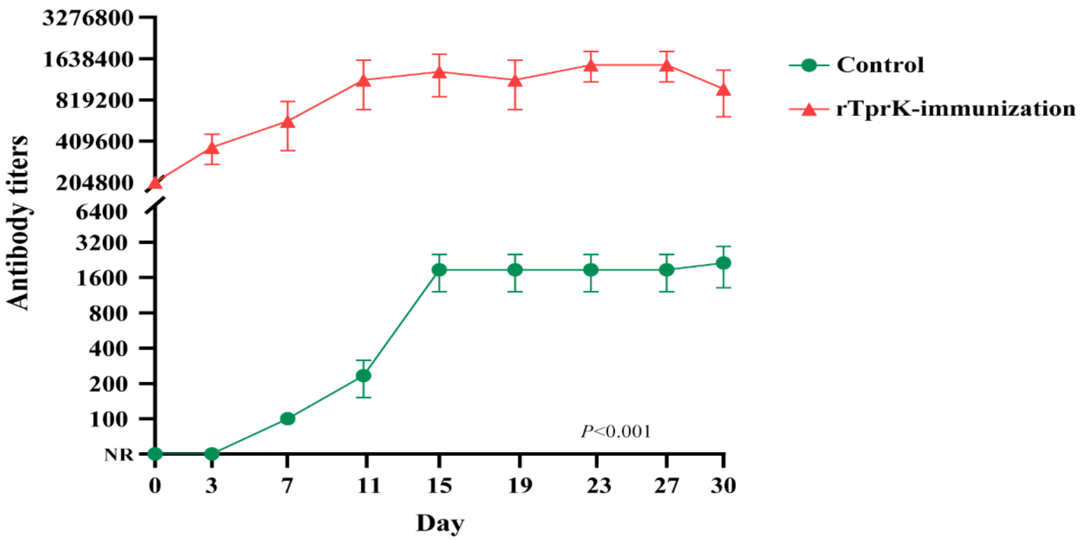

Supplementary Figure 1. The titer change of anti-rTprK antibody in the two groups after challenge with *T. pallidum*. The data are expressed as the mean ± SD. The overall antibody titer between the two groups were compared using repeated-measures ANOVA. NR, nonreactive.

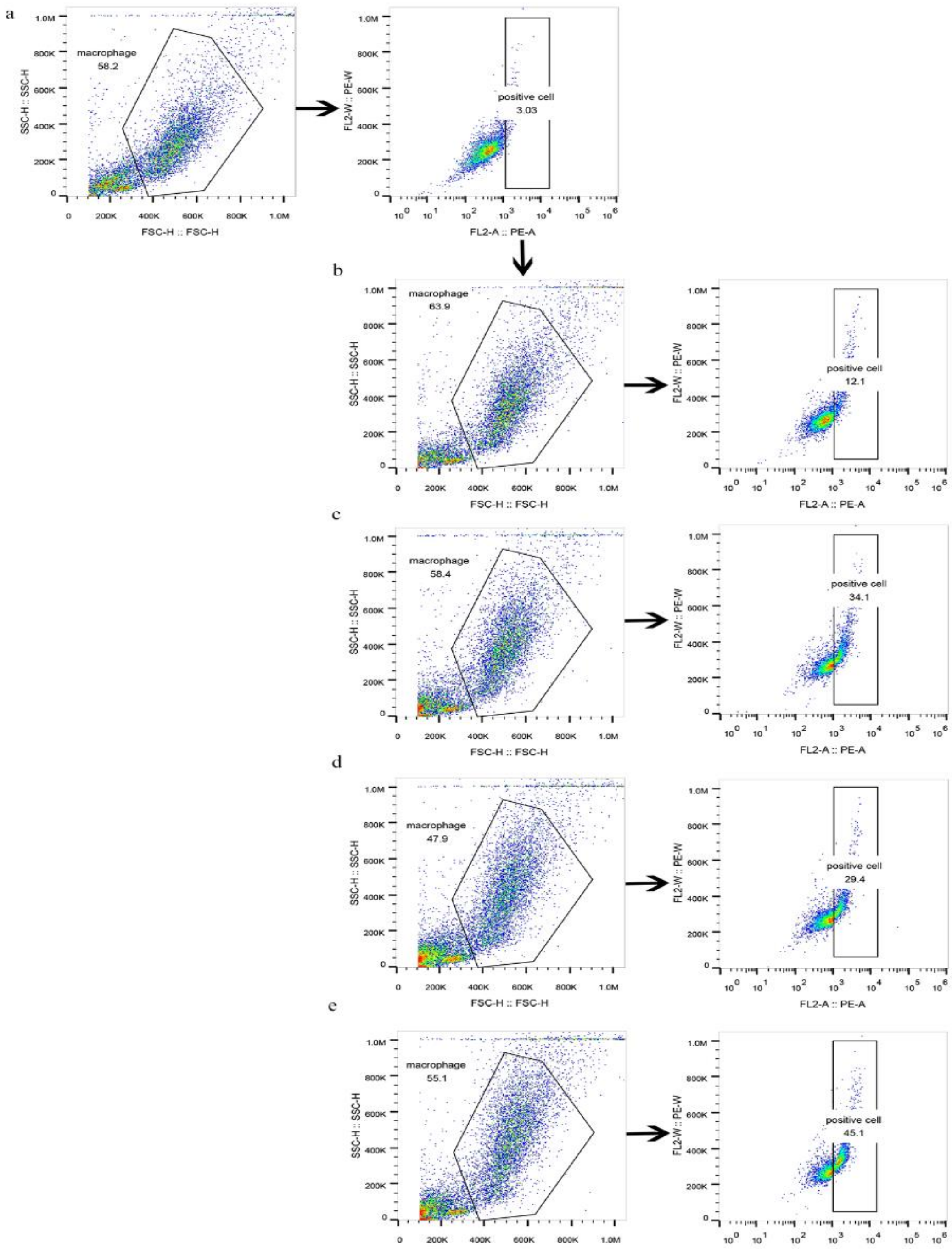

Supplementary Figure 2. Gating strategies used for cell sorting. a Gating strategy to sort negative *T. pallidum*-internalization macrophages. **b, c** Gating strategy to sort positive *T. pallidum*-internalization macrophages with prechallenge rabbit sera from control and rTprK-immunized rabbits. **d, e** Gating strategy to sort positive *T. pallidum*-internalization macrophages with postchallenge rabbit sera from control and rTprK-immunized rabbits.

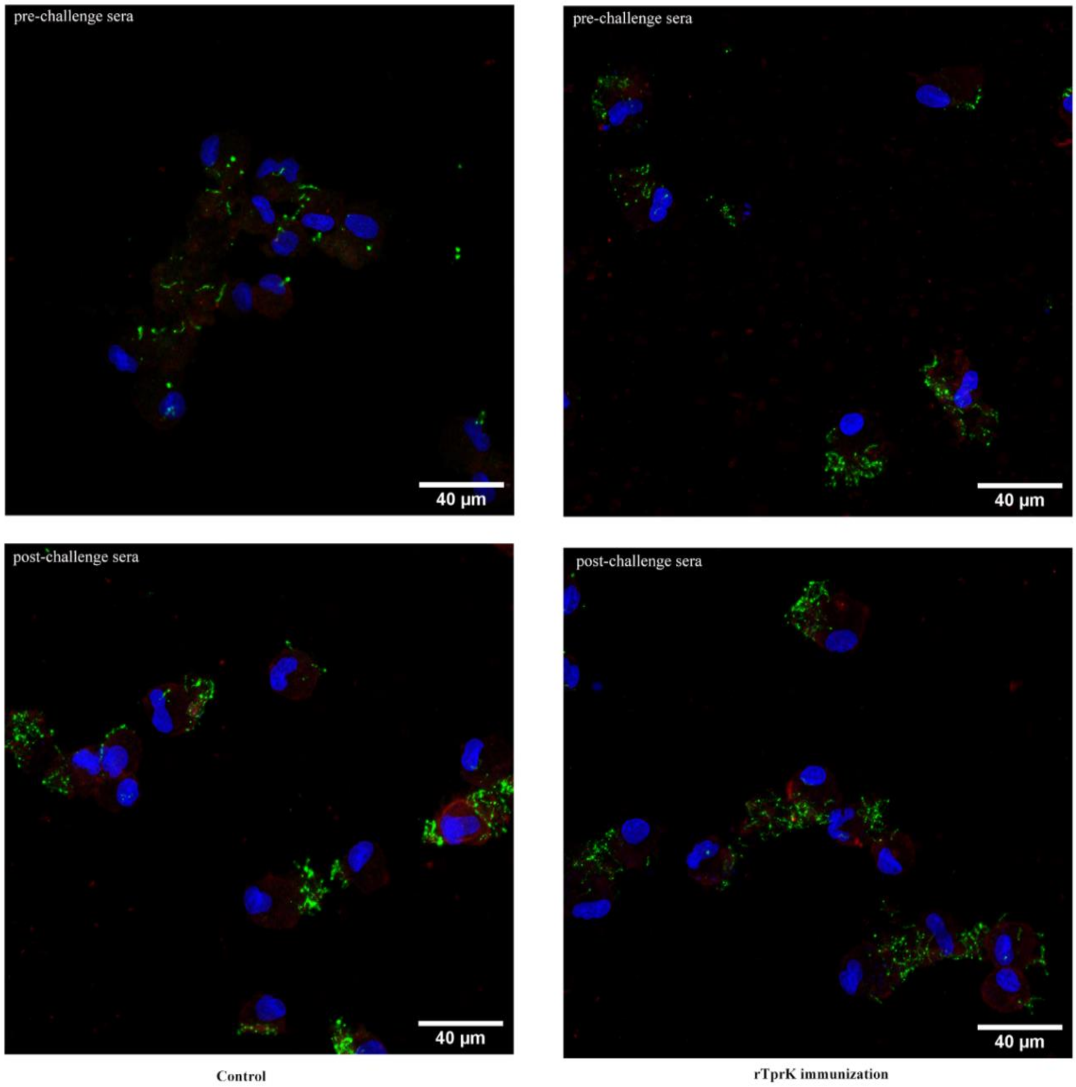

Supplementary Figure 3. Phagocytosis of macrophages by indirect immunofluorescence analysis. Scale bars were shown as 40 µm.

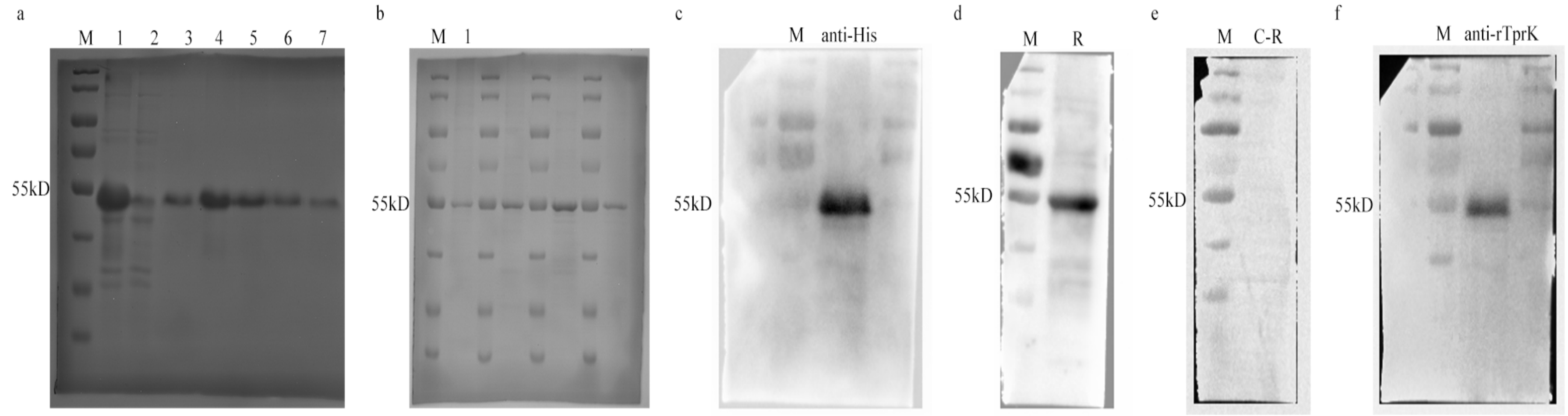

Supplementary Figure 4. Confirmation of the recombinant full-length TprK protein. a SDS–PAGE analysis of fractions following nickel affinity chromatography. M: MW markers (kDa); lane #1: pellet of the cell lysate; lane #2: flow-through; lane #3～7: elution fraction using 1 M imidazole. **b** M: MW markers (kDa); lane #1: purified full-length rTprK protein. **c** Western blot analysis of rTprK with anti-His monoclonal antibody (anti-His). **d** Western blot analysis of rTprK with sera from rabbits infected with the *T. pallidum* Nichols strain (R). **e** Western blot analysis of rTprK with sera from normal rabbits (C-R). f Western blot analysis of TprK protein in Nichols whole cell lysates with sera from the rTprK-immunized rabbits (anti-rTprK).

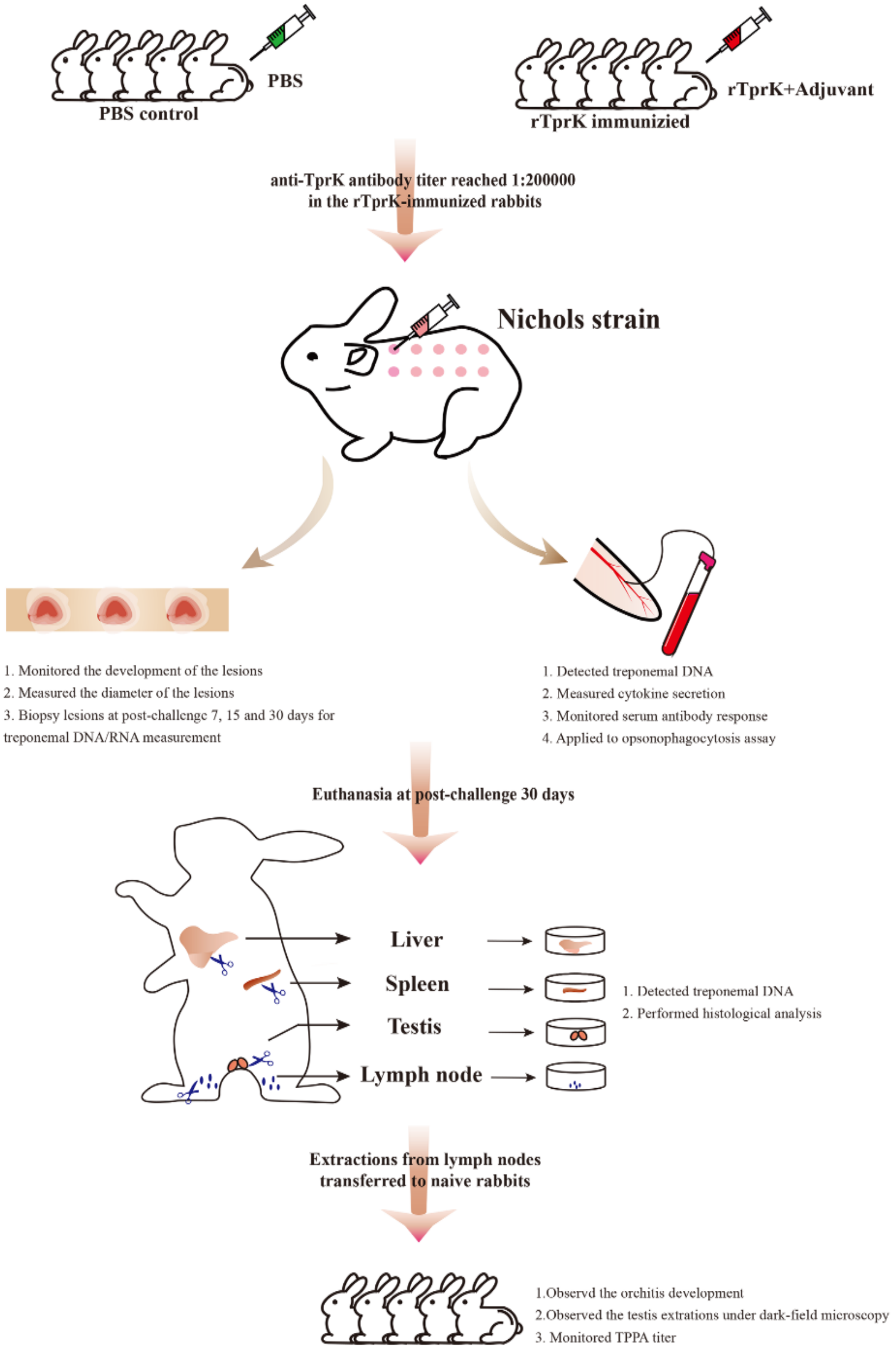

Supplementary Figure 5. Overview of the immunization and challenge infection procedure

Supplementary Table1. Results of the rabbit infectivity test

	Seroconversion (Days, mean ± SD)	Orchitis (N, %)	Positive darkfield microscopy (N, %)	RIT
rTprK-immunization	43.2±3.3	0/5 (0%)	1/5 (20%)	Positive
Control	25.6±2.2	3/5 (60%)	5/5 (100%)	Positive
P value	<0.001	>0.05	<0.05	
