# Supplementary material for: Insights into the protective immune response by immunization with full-length recombinant TprK protein: cellular and humoral responses
Source: NPJ Vaccines. 2023 Sep 29;8:146. doi: 10.1038/s41541-023-00748-1 (PMC10542339; doi:10.1038/s41541-023-00748-1)
Supplement: Supplementary file 2 — reporting-summary [file 41541_2023_748_MOESM2_ESM.pdf]

nature portfolio

Corresponding author(s): Tan-Ci Yang

Last updated by author(s): Sep 5, 2023

Reporting Summary

Nature Portfolio wishes to improve the reproducibility of the work that we publish. This form provides structure for consistency and transparency in reporting. For further information on Nature Portfolio policies, see our Editorial Policies and the Editorial Policy Checklist.

Statistics

For all statistical analyses, confirm that the following items are present in the figure legend, table legend, main text, or Methods section.

n/a	Confirmed	
☐	☒	The exact sample size (*n*) for each experimental group/condition, given as a discrete number and unit of measurement
☐	☒	A statement on whether measurements were taken from distinct samples or whether the same sample was measured repeatedly
☐	☒	The statistical test(s) used AND whether they are one- or two-sided<br>*Only common tests should be described solely by name; describe more complex techniques in the Methods section.*
☐	☒	A description of all covariates tested
☐	☒	A description of any assumptions or corrections, such as tests of normality and adjustment for multiple comparisons
☐	☒	A full description of the statistical parameters including central tendency (e.g. means) or other basic estimates (e.g. regression coefficient) AND variation (e.g. standard deviation) or associated estimates of uncertainty (e.g. confidence intervals)
☐	☒	For null hypothesis testing, the test statistic (e.g. *F*, *t*, *r*) with confidence intervals, effect sizes, degrees of freedom and *P* value noted<br>*Give P values as exact values whenever suitable.*
☐	☒	For Bayesian analysis, information on the choice of priors and Markov chain Monte Carlo settings
☐	☒	For hierarchical and complex designs, identification of the appropriate level for tests and full reporting of outcomes
☐	☒	Estimates of effect sizes (e.g. Cohen's *d*, Pearson's *r*), indicating how they were calculated

Our web collection on statistics for biologists contains articles on many of the points above.

Software and code

Policy information about availability of computer code

Data collection	no software was used
Data analysis	no software was used

For manuscripts utilizing custom algorithms or software that are central to the research but not yet described in published literature, software must be made available to editors and reviewers. We strongly encourage code deposition in a community repository (e.g. GitHub). See the Nature Portfolio guidelines for submitting code & software for further information.

Data

Policy information about availability of data

All manuscripts must include a data availability statement. This statement should provide the following information, where applicable:

- Accession codes, unique identifiers, or web links for publicly available datasets
- A description of any restrictions on data availability
- For clinical datasets or third party data, please ensure that the statement adheres to our policy

The sequence of full-length tprK gene used for recombinant protein product was deposited in GenBank under accession number OR233723. The authors declare that all data supporting the findings of this study are available within the paper and its supplementary information files.

Research involving human participants, their data, or biological material

Policy information about studies with human participants or human data. See also policy information about sex, gender (identity/presentation), and sexual orientation and race, ethnicity and racism.

Reporting on sex and gender	The research did not involve human participants.
Reporting on race, ethnicity, or other socially relevant groupings	The research did not involve human participants.
Population characteristics	The research did not involve human participants.
Recruitment	The research did not involve human participants.
Ethics oversight	The research did not involve human participants.

Note that full information on the approval of the study protocol must also be provided in the manuscript.

Field-specific reporting

Please select the one below that is the best fit for your research. If you are not sure, read the appropriate sections before making your selection.

☒ Life sciences ☐ Behavioural & social sciences ☐ Ecological, evolutionary & environmental sciences

For a reference copy of the document with all sections, see nature.com/documents/nr-reporting-summary-flat.pdf

Life sciences study design

All studies must disclose on these points even when the disclosure is negative.

Sample size	No sample-size calculation was performed. The study aimed to evaluate the immunogenicity of full-length rTprK in the rabbit model of syphilis infected with the Nichols strain. The number of rabbits selected for the experiment was based on the previous relevant studies on the syphilis vaccine. And at least three rabbits were included in each group for the results to be statistically significant. This study contained 10 rabbits for the experiment, and each group contained five rabbits. The samples size was sufficient to the next analysis.
Data exclusions	No data was excluded.
Replication	Each sample was analyzed in parallel with the duplicate wells. All attempts at replication were successful.
Randomization	Male New Zealand White rabbits (3.5 kg, 13-15 weeks of age, Xiamen University Laboratory Animal Center, Xiamen, China) with negative Venereal Disease Research Laboratory (VDRL) test and T. pallidum particle agglutination (TPPA) test were randomly selected in this study.
Blinding	The grouping of rabbits in this study was blinded to both observers and experimenters, and only the program administrators were aware of the grouping of specific rabbits.

Reporting for specific materials, systems and methods

We require information from authors about some types of materials, experimental systems and methods used in many studies. Here, indicate whether each material, system or method listed is relevant to your study. If you are not sure if a list item applies to your research, read the appropriate section before selecting a response.

Materials & experimental systems

n/a	Involved in the study	
☐	☒	Antibodies
☐	☐	Eukaryotic cell lines
☐	☐	Palaeontology and archaeology
☐	☒	Animals and other organisms
☐	☐	Clinical data
☐	☐	Dual use research of concern
☐	☐	Plants

Methods

n/a	Involved in the study	
☒	☐	ChIP-seq
☐	☒	Flow cytometry
☒	☐	MRI-based neuroimaging

Antibodies

Antibodies used	anti-Tp47 mouse monoclonal antibody
Validation	The antibody is for Treponema pallidum, which was validated by Chen et al. (The whole process of macrophage-Treponema pallidum interactions: Opsonic phagocytosis, nonopsonic phagocytosis and active invasion. International immunopharmacology 107, 108657 (2022))

Eukaryotic cell lines

Policy information about cell lines and Sex and Gender in Research

Cell line source(s)	*State the source of each cell line used and the sex of all primary cell lines and cells derived from human participants or vertebrate models.*
Authentication	*Describe the authentication procedures for each cell line used OR declare that none of the cell lines used were authenticated.*
Mycoplasma contamination	*Confirm that all cell lines tested negative for mycoplasma contamination OR describe the results of the testing for mycoplasma contamination OR declare that the cell lines were not tested for mycoplasma contamination.*
Commonly misidentified lines (See ICLAC register)	*Name any commonly misidentified cell lines used in the study and provide a rationale for their use.*

Palaeontology and Archaeology

Specimen provenance	*Provide provenance information for specimens and describe permits that were obtained for the work (including the name of the issuing authority, the date of issue, and any identifying information). Permits should encompass collection and, where applicable, export.*
Specimen deposition	*Indicate where the specimens have been deposited to permit free access by other researchers.*
Dating methods	*If new dates are provided, describe how they were obtained (e.g. collection, storage, sample pretreatment and measurement), where they were obtained (i.e. lab name), the calibration program and the protocol for quality assurance OR state that no new dates are provided.*

☐ Tick this box to confirm that the raw and calibrated dates are available in the paper or in Supplementary Information.

Ethics oversight	*Identify the organization(s) that approved or provided guidance on the study protocol, OR state that no ethical approval or guidance was required and explain why not.*

Note that full information on the approval of the study protocol must also be provided in the manuscript.

Animals and other research organisms

Policy information about studies involving animals; ARRIVE guidelines recommended for reporting animal research, and Sex and Gender in Research

Laboratory animals	Male New Zealand White rabbits (3.5 kg, 13-15 weeks of age, Xiamen University Laboratory Animal Center, Xiamen, China) with negative Venereal Disease Research Laboratory (VDRL) test and T. pallidum particle agglutination (TPPA) test were randomly selected in this study.
Wild animals	The study did not involve wide animals.
Reporting on sex	Male
Field-collected samples	Rabbits were housed individually at 15 to 18°C and given antibiotic-free food and water.
Ethics oversight	This study was approved by the Institutional Ethics Committee of Zhongshan Hospital of Xiamen University, School of Medicine, Xiamen University. All rabbit experimental protocols were approved by the Animal Experimental Ethics Committee of the School of Medicine, Xiamen University.

Note that full information on the approval of the study protocol must also be provided in the manuscript.

Clinical data

Policy information about clinical studies

All manuscripts should comply with the ICMJE guidelines for publication of clinical research and a completed CONSORT checklist must be included with all submissions.

Clinical trial registration	*Provide the trial registration number from ClinicalTrials.gov or an equivalent agency.*
Study protocol	*Note where the full trial protocol can be accessed OR if not available, explain why.*
Data collection	*Describe the settings and locales of data collection, noting the time periods of recruitment and data collection.*
Outcomes	*Describe how you pre-defined primary and secondary outcome measures and how you assessed these measures.*

Dual use research of concern

Policy information about dual use research of concern

Hazards

Could the accidental, deliberate or reckless misuse of agents or technologies generated in the work, or the application of information presented in the manuscript, pose a threat to:

No	Yes	
☐	☐	Public health
☐	☐	National security
☐	☐	Crops and/or livestock
☐	☐	Ecosystems
☐	☐	Any other significant area

Experiments of concern

Does the work involve any of these experiments of concern:

No	Yes	
☐	☐	Demonstrate how to render a vaccine ineffective
☐	☐	Confer resistance to therapeutically useful antibiotics or antiviral agents
☐	☐	Enhance the virulence of a pathogen or render a nonpathogen virulent
☐	☐	Increase transmissibility of a pathogen
☐	☐	Alter the host range of a pathogen
☐	☐	Enable evasion of diagnostic/detection modalities
☐	☐	Enable the weaponization of a biological agent or toxin
☐	☐	Any other potentially harmful combination of experiments and agents

Flow Cytometry

Plots

Confirm that:

☒ The axis labels state the marker and fluorochrome used (e.g. CD4-FITC).

☒ The axis scales are clearly visible. Include numbers along axes only for bottom left plot of group (a 'group' is an analysis of identical markers).

☒ All plots are contour plots with outliers or pseudocolor plots.

☒ A numerical value for number of cells or percentage (with statistics) is provided.

Methodology

Sample preparation	Proteose peptone-induced rabbit peritoneal macrophages were isolated from normal rabbits with negative VDRL test and TPPA test. Macrophages were cultured in DMEM medium with 10% serum, and fresh T. pallidum was added at an MOI of 10:1 for 12 h of incubation. Macrophages were trypsinized and collected by centrifugation. 5% BSA was added to block the cells for 1 h. The cells were collected and resuspended with FIX PERM MEDIUM A, then were incubated at 4°C for 15 min and were washed thrice with DMEM medium. Subsequently, FIX PERM MEDIUM B and Anti-Tp47 mouse monoclonal antibody

were added, incubated at 4°C for 20 min, and washed thrice with DMEM medium. Finally, phycoerythrin (PE)-conjugated anti-mouse secondary antibody was added, incubated at 4°C for 20 min, and washed thrice with DMEM medium. The cells were resuspended with PBS for flow cell analysis.

Instrument	FACS Canto II flow cytometer (BD, New Jersey, USA)
Software	FlowJo7.6.4 software (Tree Star, Ashland, OR, USA).
Cell population abundance	The abundance of the relevant populations (macrophages) was appropriate at 50%, which was determined by FSC and SSC gates.
Gating strategy	The gating strategy designed was mainly depending on the setting of Isotype control. By using the isotype control, the boundary for the negative staining cells was defined. Then the cells whose fluorescent signal is above this boundary were defined as the positive staining cells.

☒ Tick this box to confirm that a figure exemplifying the gating strategy is provided in the Supplementary Information.
